# Electrical stimulation mapping in the medial prefrontal cortex induced auditory hallucinations of episodic memory: A case report

**DOI:** 10.3389/fnhum.2022.815232

**Published:** 2022-07-28

**Authors:** Qiting Long, Wenjie Li, Wei Zhang, Biao Han, Qi Chen, Lu Shen, Xingzhou Liu

**Affiliations:** ^1^Key Laboratory of Brain, Cognition and Education Sciences, Ministry of Education, Guangzhou, China; ^2^Guangdong Key Laboratory of Mental Health and Cognitive Science, Center for Studies of Psychological Application, School of Psychology, South China Normal University, Guangzhou, China; ^3^Department of Neurology, Beijing Tsinghua Changgung Hospital, Beijing, China

**Keywords:** electrical stimulation mapping, auditory processing, episodic memory retrieval, default mode network, high gamma activity, case report

## Abstract

It has been well documented that the auditory system in the superior temporal cortex is responsible for processing basic auditory sound features, such as sound frequency and intensity, while the prefrontal cortex is involved in higher-order auditory functions, such as language processing and auditory episodic memory. The temporal auditory cortex has vast forward anatomical projections to the prefrontal auditory cortex, connecting with the lateral, medial, and orbital parts of the prefrontal cortex. The connections between the auditory cortex and the prefrontal cortex thus help in localizing, recognizing, and comprehending external auditory inputs. In addition, the medial prefrontal cortex (MPFC) is believed to be a core region of episodic memory retrieval and is one of the most important regions in the default mode network (DMN). However, previous neural evidence with regard to the comparison between basic auditory processing and auditory episodic memory retrieval mainly comes from fMRI studies. The specific neural networks and the corresponding critical frequency bands of neuronal oscillations underlying the two auditory functions remain unclear. In the present study, we reported results of direct cortical stimulations during stereo-electro-encephalography (SEEG) recording in a patient with drug-resistant epilepsy. Electrodes covered the superior temporal gyrus, the operculum and the insula cortex of bilateral hemispheres, the prefrontal cortex, the parietal lobe, the anterior and middle cingulate cortex, and the amygdala of the left hemisphere. Two types of auditory hallucinations were evoked with direct cortical stimulations, which were consistent with the habitual seizures. The noise hallucinations, i.e., “I could hear buzzing noises in my head,” were evoked with the stimulation of the superior temporal gyrus. The episodic memory hallucinations “I could hear a young woman who was dressed in a red skirt saying: What is the matter with you?,” were evoked with the stimulation of MPFC. The patient described how she had met this young woman when she was young and that the woman said the same sentence to her. Furthermore, by analyzing the high gamma power (HGP) induced by direct electrical stimulation, two dissociable neural networks underlying the two types of auditory hallucinations were localized. Taken together, the present results confirm the hierarchical processing of auditory information by showing the different involvements of the primary auditory cortex vs. the prefrontal cortex in the two types of auditory hallucinations.

## Introduction

The temporal auditory cortex, including the superior temporal gyrus and the sulcus, is divided into the core region, the belt region, the para-belt region, and the rostral superior temporal gyrus (Rauschecker et al., 1995, 1997; Rauschecker and Tian, 2004; Romanski and Averbeck, 2009). The prefrontal lobe is involved in various higher-order cognitive functions, such as decision-making, motor planning, and communication (Barbas, 1995; Petrides, 2005; Fuster, 2008; Kostopoulos and Petrides, 2008). The temporal auditory cortex has vast forward anatomical projections to the prefrontal auditory cortex, connecting with the lateral, medial, and orbital parts of the prefrontal cortex (Petrides and Pandya, 1988; Barbas et al., 1999; Romanski et al., 1999; Romanski and Goldman-Rakic, 2002; Romanski and Averbeck, 2009; Plakke and Romanski, 2014). Conversely, the prefrontal cortex has backward anatomical projections with the temporal auditory cortex (Barbas et al., 2011). The connections between the auditory cortex and the prefrontal cortex are involved in localizing, recognizing, and comprehending external auditory inputs. In addition, the vast and diverse interconnections between the temporal auditory cortex and the prefrontal cortex suggest a close relationship between auditory information processing and prefrontal functions. The temporal auditory cortex inputs all levels of sound information to the prefrontal cortex. Our brain processes the relevant acoustic information and ignores the irrelevant noises and further transforms the selected auditory information into spatial locations and semantics for object recognition, communication, and motor execution. Therefore, the prefrontal cortex is regarded as a higher-order “auditory field” in addition to the temporal cortex.

In addition, the medial prefrontal cortex (MPFC) plays an important role in social cognition and self-referential cognition. It is believed to be a core region during episodic memory retrieval, especially in the retrieval of autobiographical information (Svoboda et al., 2006; McDermott et al., 2009; Eichenbaum, 2017). Therefore, the prefrontal cortex involves dealing with memory retrieval with sounds. In an fMRI study, regions, such as the MPFC, the cingulate cortex, and the medial temporal structures, were associated with enhanced activity in the general recollection network (Rugg and Vilberg, 2013). Furthermore, MPFC is one of the major regions in the Default Mode Network (DMN) (Sestieri et al., 2011). The DMN includes three main regions: The MPFC, the posterior cingulate cortex, and the bilateral angular gyrus, and it can be divided into many sub-systems, including episodic memory and self-referential cognition (Andrews-Hanna et al., 2010). Previous studies pointed out that DMN is responsible for episodic memory retrieval (Buckner et al., 2008; Foster et al., 2012), and the frontoparietal network is also involved in episodic memory retrieval (St Jacques et al., 2011; Westphal et al., 2017). However, previous neural evidence with regard to the comparison between basic auditory processing and auditory episodic memory retrieval mainly comes from fMRI studies.

One optimal method to examine the functional roles of given regions in basic auditory processing and auditory episodic memory retrieval is direct electrical stimulation, in which a volley of electrical charge is delivered to a focal brain area to perturb its function (Selimbeyoglu and Parvizi, 2010). In the present study, we induced two different types of auditory hallucinations by applying electrical stimulation mapping (ESM) to an epilepsy patient who was undergoing stereo-electro-encephalography (SEEG) recording with implanted electrodes. One type of auditory hallucination was noise hallucination with only basic sound during stimulation of the superior temporal gyrus. The other type was an auditory hallucination that contained semantic auditory hallucinations and episodic memory retrieval during the stimulation of the MPFC. It has been suggested that the functional changes induced by electrical stimulation may reflect the dysfunction of a large-scale network but not just one site (Mandonnet et al., 2010,^2016^). Therefore, in the present study, we aimed to identify the brain regions and networks associated with the two different auditory hallucinations.

It has been demonstrated that high gamma activity was extensively recorded in acoustic and speech processing (Edwards et al., 2005; Chang et al., 2011; Nourski et al., 2014), auditory verbal memory (Kaiser et al., 2003), and auditory language comprehension (Nakai et al., 2017; Ikegaya et al., 2019). A recent study showed that high-frequency activity induced by 50 Hz electrical stimulation during language disturbances may help to localize language-related regions (Perrone-Bertolotti et al., 2020). In the present study, we used a similar approach to identify brain areas and networks associated with two different auditory hallucinations by analyzing the high gamma band activity induced by 50 Hz electrical stimulation. We found that the temporal auditory network was primarily involved in the noise hallucinations, while the DMN and frontoparietal network were involved in the episodic memory hallucinations. These results shed light on the fact that the prefrontal cortex is involved in the auditory information processing and retrieval of episodic memory. These results were consistent with previous studies that MPFC is involved in the dual auditory processing networks and the episodic memory retrieval networks, such as DMN.

## Materials and methods

### Subject

The subject was a 30-year-old female patient implanted with intracranial electrodes to localize the source of drug-resistant seizures who provided informed consent to participate in this study. The procedure was approved by the Ethics Committee of the School of Psychology, South China Normal University. The patient also consented to the publication of the present case report. Detailed history, neurological examination, neuropsychological evaluation, neuroimaging, high-resolution structural magnetic resonance imaging, and positron emission tomography/computed tomography (PET/CT) were accessed during the non-invasive diagnostic evaluation. Detailed profiles of the patient are provided in Supplementary Table 1. The habitual seizure semiology was: aura (including rustling of leaves, or the voice of a young woman who was dressed in a red skirt saying **“**what is the matter with you**”**) → chapeau de gendarme → automatic movement (hands) → hyperventilation. The duration of the seizure was about 10–30 s. In the first few years, the seizure frequency was 1–2 times a year, but it became more and more frequent, although, with appropriate and adequate anti-epileptic drugs, it became 3–10 times per day prior to surgery. There was no relevant family history of the patient. Additionally, she did not have any intracranial surgeries before SEEG implantation. After the invasive evaluation with SEEG, the epileptic zone was identified, and a tailored resection was made. The patient has been seizure-free for 3 years since the surgical treatment.

### Intracranial implantation

During the pre-surgical SEEG evaluation, the clinical team gave a detailed and precise diagnosis of the anatomical location of the epileptogenic zone and its relationship with the eloquent cortex. The patient was implanted with 16 electrodes (left: 13 and right: 3). The depth electrodes were semi-rigid platinum/iridium with either 7 or 18 contacts (2 mm in length, 0.8 mm in diameter, and 1.5 mm apart). The distribution of all the electrodes in the brain is shown in Supplementary Figure 1. To locate the electrode contacts, a CT scan after the electrode implantation and a pre-operative MRI were co-registered in FSL,^1^ and then, electrode coordinates were obtained using FreeSurfer scripts (FreeSurfer scripts:).^2^ The electrodes were positioned in different areas based on the Destrieux Atlas (Destrieux et al., 2010).

### Electrical stimulation mapping

The ESM was performed by a professional physician during the SEEG recording. SEEG was recorded with simultaneous video recording, using a 256-channel Nihon Kohden Neurofax 1200A Digital System. Bipolar electrical stimuli (50 Hz, pulse width 0.3 ms, intensity 0.6–5 mA, duration 3–5 s) were delivered by means of biphasic rectangular stimuli of alternating polarity. The patient was not informed of the stimulation onset/offset and was asked to describe any feelings she experienced. Both subjective reports of her and objective observations from the physician were noted down on the patient’s medical record, which were ultimately reviewed on recording and confirmed by two neurologists and a neuroscientist.

### Localization of responsive electrodes

Recording electrodes were considered to be responsive if HGP was within the post-stimulation period (0.2–2.8 s) against (*z* > 2) the pre-stimulation baseline period (–500 to –20 ms). HGP was computed by means of a continuous wavelet transform of data for the frequency range from 70 to 160 Hz. The length of the wavelets increased linearly from 10 cycles at 70 Hz to 26 cycles at 160 Hz. The wavelet analysis was performed with a Morlet wavelet by applying a Gaussian-shaped taper. The spectral resolution was chosen at 2 Hz. The start of the stimulation onset was based on the stimulation artifact. Then, the high gamma band power within the post-stimulation period was transformed into a *z*-score against the pre-stimulation baseline period in each stimulation condition. To eliminate any influence of stimulation artifacts in the predicted SEEG power changes, we masked out *z*-values above 10 along the frequency dimension and removed channels (less than 15 mm away from the stimulating electrodes). Bad recording electrodes were excluded from all analyses. Finally, to obtain responsive electrodes within each type of auditory hallucination, response electrodes under all relevant stimulation conditions were intersected.

### High gamma power *z*-scores of electrodes within the networks of interest

**FIGURE 1:**
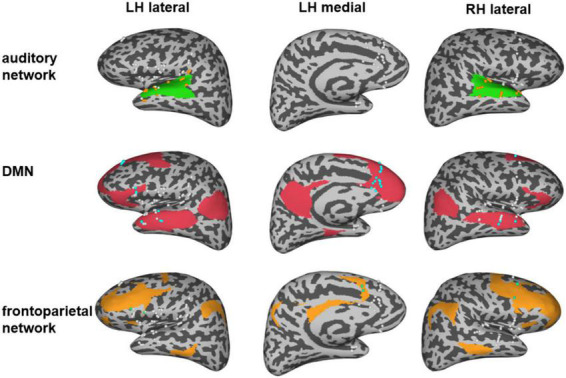
Distribution of electrodes in three different networks. The green region represents the auditory network and the orange electrodes indicate the electrodes in the auditory network. The red region represents the default mode network (DMN), and the blue electrodes indicate the electrodes in the DMN. The orange region represents the frontal network and the green electrodes indicate the electrodes in the frontal network. Note that the white electrodes represent electrodes that were implanted outside the corresponding network.

All implanted electrodes (except electrodes in the epileptic zone, as seen in Supplementary Figure 1) were categorized into DMN and frontoparietal networks using Yeo Atlas (Yeo et al., 2011) and categorized into the auditory network (early auditory cortex and auditory association cortex) using the multimodal cortical parcellation (Glasser et al., 2016). The early auditory areas include A1, Lateral Belt (LBelt), Medial Belt (MBelt), Para-Belt (PBelt), and the retro-insular cortex (RI). The auditory association cortex includes A4, A5, STSdp, STSda, STSvp, STSva, STGa, and TA2. Electrodes of the MPFC within DMN were defined using the multimodal cortical parcellation, including a32pr, d32, and 9 m (Glasser et al., 2016). All these automatic labels were confirmed visually by two neurologists (anatomical locations of all network electrode sites as shown in Figure 1). The HGP z-scores of the recording electrodes in each network within each type of auditory hallucination were determined by averaging the HGP *z*-values from relevant stimulation conditions.

### Statistical analyses

To examine whether the HGP *z*-values of a specific network showed a statistical difference between noise hallucinations and episodic memory hallucinations conditions, we performed a paired *t*-test for each network and corrected for multiple comparisons with the false discovery correction (FDR) procedure (Benjamini and Hochberg, 1995).

## Results

### Electrical stimulation in different sites induced different hallucinations symptoms

**FIGURE 2:**
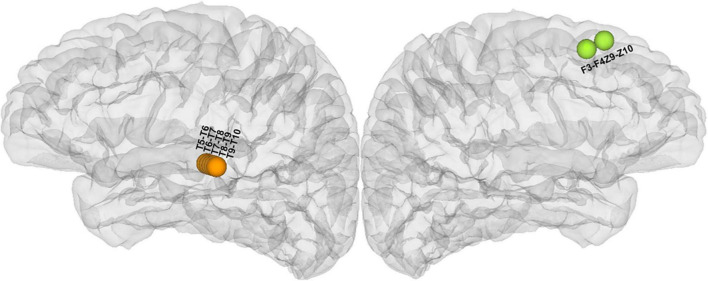
Anatomical location of all cortical stimulated sites responsible for hallucinations during electrical stimulation mapping (ESM) (the orange electrodes in the lateral view of the left hemisphere were responsible for noise hallucinations and the green electrodes in the medial view of the left hemisphere were responsible for episodic memory hallucinations).

Electrical stimulation mapping could evoke a summation effect in a vast volume of the cortex and cause a transient experience or changes in behavior. The subjective experiences or behavioral changes induced by ESM in the patient could be categorized into two main types of auditory hallucinations: noise hallucinations vs. episodic memory hallucinations. Noise hallucinations were described by the patient as “I could hear buzzing noises in my head.” The episodic memory hallucinations were complex auditory hallucinations with specific contents, i.e., the patient reported that “I could hear a young woman’s voice saying: what is the matter with you?” The latter type of auditory aura was her habitual seizure. The patient further described that she seemed to see her when she heard this voice. She had seen this girl before, wearing a red dress, and had said the same thing to her. Therefore, we believe that she recalled episodic memories during the stimulation. The noise hallucinations were evoked on the middle part of the superior temporal gyrus of the left hemisphere (Figure 2 and Supplementary Table 2) while the episodic memory hallucinations symptoms were elicited on the left superior frontal gyrus (Figure 2 and Supplementary Table 2) *via* ESM.

### High gamma activity associated with hallucinations symptoms

**FIGURE 3:**
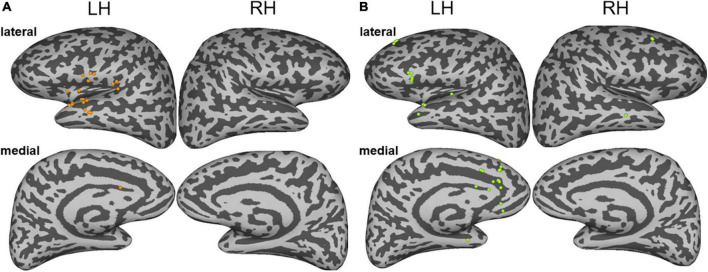
**(A)** The sites of stimulation-induced high gamma-band power during noise hallucinations. **(B)** The sites of stimulation-induced high gamma-band power during episodic memory hallucinations. Lateral and medial views of the left and right hemispheres are represented.

The current applied to the stimulation sites not only affects the local activity but also affects the network areas connected with the stimulation sites (Perrone-Bertolotti et al., 2020). Therefore, the subjective experiences and behavioral changes elicited by the electrical stimulations were attributed to the functional changes of the large-scale distributed neural networks: both the noise and the episodic memory hallucinations during the ESM could be explained by large-scale brain regions or networks. To identify the brain regions related to different auditory hallucinations, we analyzed the changes in high gamma band activity, which has been considered a potential specific biological support of auditory processing (Edwards et al., 2005; Chang et al., 2011; Nourski et al., 2014). We identified brain regions in which the high gamma band power is higher than the baseline activity (*z* > 2) (as shown in detail in the “Materials and methods” section). The results showed that the two types of auditory hallucinations were associated with increased evoked high gamma activity in different responsive regions. Specifically, the noise hallucinations were associated with enhanced high gamma activity in the ventral auditory stream, including the superior temporal gyrus, the Heschel’s gyrus, the central operculum, the parietal operculum, the frontal operculum, the anterior insula, and the cingulate electrodes (Figure 3A). Among these regions, the superior temporal gyrus and the middle temporal gyrus were the most representative regions, with 11 electrodes having enhanced high gamma activity, accounting for 38% of all response contacts. These representative regions are key regions of the auditory network. However, episodic memory hallucinations were associated with the dorsal MPFC, the cingulate gyrus, the pars triangularis, the superior temporal gyrus, and the middle temporal gyrus (Figure 3B). Especially, the medial frontal cortex, the cingulate gyrus, and the inferior frontal gyrus were representative regions, with 19 electrodes having enhanced high gamma activity, accounting for 61% of all responsive electrodes. These representative regions are key regions of the DMN and the frontoparietal network.

### Functional networks involved in different hallucinations symptoms

**FIGURE 4:**
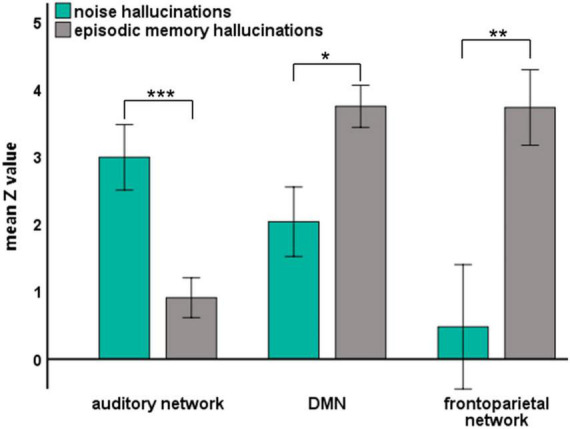
The mean HGP *z*-values of each auditory hallucinations condition in three brain networks. Significant group differences were denoted with asterisks (error bars indicate ± 1 SEM, *p < 0.05, **p < 0.01, and ***p < 0.001).

By analyzing the responsive brain areas associated with specific hallucinations, we found that the occurrence of the two hallucinations involved distinct regions, suggesting that the two hallucinations may be involved in different networks. Previous neural evidence suggested important roles of the auditory network, the frontoparietal network (St Jacques et al., 2011; Westphal et al., 2017), and the DMN (Buckner et al., 2008; Foster et al., 2012) in auditory processing and episodic memory retrieval. Therefore, we further directly confirmed the different roles of these different networks in noise and episodic memory hallucinations. We reanalyzed data from another perspective: We assigned all implanted electrodes (except the electrodes in the epileptic zone) into different functional networks of interest (as shown in Figure 1). Then, we investigated the HGP difference between noise and episodic memory hallucinations within each network. For the auditory network, mean HGP *z*-values in the noise hallucination condition were higher than that in the episodic memory hallucination condition [*t*_(35)_ = 4.072, *p* < 0.001, FDR corrected]. However, for the DMN [*t*_(31)_ = –2.480, *p* < 0.05, FDR corrected] and the frontoparietal network [*t*_(9)_ = –5.192, *p* < 0.01, FDR corrected], mean HGP *z*-values in the episodic memory hallucination condition were significantly higher than that in the noise hallucination condition (Figure 4).

Since the MPFC in the DMN has been considered a core region in episodic memory retrieval, to further directly show the role of the MPFC in episodic memory hallucinations, we divided the electrodes of the DMN into the MPFC and the others (as shown in section “Materials and Methods”) and then analyzed the HGP difference between the two hallucinations for the different groups of DMN electrodes. For the electrodes of the MPFC within the DMN, the mean HGP *z*-values in the episodic memory hallucination condition were significantly higher than in the noise hallucination condition [*t*_(11)_ = 3.365, *p* < 0.01], while for electrodes within the DMN but outside the MPFC, there was no significant HGP difference between the two hallucination conditions [*t*_(19)_ = 0.728, *p* = 0.48] (as shown in Supplementary Figure 2].

## Discussion

In this case study, with the use of the direct cortical stimulation method, we analyzed high gamma activity induced by 50 Hz electrical stimulation to identify brain regions and networks associated with two different auditory hallucinations. The patient had two types of habitual auditory auras, one type was noise hallucinations and the other type was episodic memory hallucinations. Both hallucinations included auditory experience, but episodic memory hallucinations recalled the experience the patient had before. The patient not only heard the voice of a young woman saying “what is the matter with you,” but she also recalled the memory that she had seen the young woman before who was dressed in a red skirt. In contrast, the noise auditory aura was only the voices of many people (Supplementary Table 2). We found that episodic memory hallucinations were elicited in the medial prefrontal regions, and both the dorsal and ventral auditory streams were activated, including the involvement of the dorsal prefrontal cortices BA8 and BA9, the anterior cingulate cortex, the ventral lateral prefrontal cortices BA44 and BA45, and the superior temporal gyrus. The noise auditory experience was elicited in the superior temporal gyrus, and the ventral stream was activated, including the involvement of the ventral lateral prefrontal cortices BA44 and BA45, the central operculum, and the superior temporal gyrus (Figures 2, 3). The results are consistent with previous auditory pathway studies (Romanski et al., 1999, 2005; Pedersen et al., 2000; Alain et al., 2001; Romanski and Goldman-Rakic, 2002). First, the auditory processing is hierarchically organized such that the “rustling of leaves” is induced in the auditory core region, and the “buzz voice” noise hallucinations were induced in the belt or the parabelt auditory region. Second, when the auditory hallucinations were involved in semantic processing, the ventral stream was activated. In this case, the ventral auditory stream in episodic memory hallucinations was activated, suggesting that this stream is involved in processing episodic auditory hallucinations.

The difference between these two auras may be due to the medial and dorsal lateral prefrontal network involvement, with additional involvement of BA8B, BA9, and the anterior cingulate cortex (Figure 3). The MPFC has proved to be associated with the emotion, memory, and complex cognitive processes (Barbas et al., 2002). The anterior cingulate cortex receives higher processed auditory information from temporal auditory regions and interconnects with the prefrontal cortex to select relevant signals and suppress noise (Micheyl et al., 2007; Medalla and Barbas, 2010). The network of the cingulate-prefrontal-temporal cortical network may help in filtering auditory information in a noise environment. Therefore, in this case, the patient may hear the sentence instead of the noise hallucinations induced in temporal regions.

Our results further confirmed the different roles of the auditory network, the DMN, and the frontoparietal network in noise hallucinations and episodic memory hallucinations (Figure 4). Specifically, the auditory cortex showed higher gamma activity in noise hallucinations than episodic memory hallucinations. This is in line with previous studies showing that high gamma activity in the auditory cortex increases during auditory processing (Edwards et al., 2005; Chang et al., 2011; Nourski et al., 2014). More importantly, the DMN and the frontoparietal network showed higher gamma activity in episodic memory hallucinations than noise hallucinations. Compelling evidence supports the relationship between high gamma activity and episodic memory retrieval (Hanslmayr et al., 2016). For example, direct hippocampal recordings from patients with epilepsy showed that high gamma power increases correlation with successful memory encoding and retrieval (Long et al., 2014). Our results further suggest that increased high gamma activity in the DMN and the frontoparietal network are also associated with episodic memory retrieval. These results are also consistent with previous imaging findings demonstrating that the DMN and the frontoparietal network were involved in episodic memory retrieval (Buckner et al., 2008; Foster et al., 2012; St Jacques et al., 2011; Westphal et al., 2017). To note, the MPFC in the DMN has been considered a core region in the neocortex during the retrieval of episodic memory. Direct evidence comes from animal studies, where the performance of episodic memory tasks has been shown to be impaired by lesions or dysfunction in MPFC (DeVito and Eichenbaum, 2010; Barker et al., 2017). In the present study, we further confirmed the critical role of the MPFC in the DMN in auditory hallucinations involving episodic memory retrieval (Supplementary Figure 2).

Taken together, the present study supports hierarchical and dual pathways in auditory processing. Both the noise and episodic memory hallucinations involved the ventral stream, suggesting that the ventral stream is involved in language comprehensive processing. Besides, the stimulation of temporal auditory regions only evoked noise hallucinations without clear semantic content and without emotional expression. Moreover, the MPFC in the DMN and the dorsal frontal cortex in the frontoparietal network were associated with auditory episodic memory retrieval. These results suggest higher-level auditory processing in the prefrontal cortex and are consistent with previous studies demonstrating an essential role of the prefrontal cortex in episodic memory retrieval.

## Data availability statement

The raw data supporting the conclusions of this article will be made available by the authors, without undue reservation.

## Ethics statement

The studies involving human participants were reviewed and approved by the Ethics Committee of School of Psychology, South China Normal University. The patients/participants provided their written informed consent to participate in this study. Written informed consent was obtained from the individual(s) for the publication of any potentially identifiable images or data included in this article.

## Author contributions

QL, LS, WZ, and XL contributed to the design of the work. QL contributed to the acquisition of data. WL and LS analyzed the data. WZ, BH, XL, and QC contributed to the interpretation of data. QL and WL wrote the first draft. WZ, BH, QC, LS, and XL edited the manuscript. All authors contributed to the article and approved the submitted version.

## Footnotes

1
www.fmrib.ox.ac.uk/fsl

2
http://surfer.nmr.mgh.harvard.edu
