# Supplementary material for: Electrical stimulation mapping in the medial prefrontal cortex induced auditory hallucinations of episodic memory: A case report
Source: Front Hum Neurosci. 2022 Jul 28;16:815232. doi: 10.3389/fnhum.2022.815232 (PMC9366097; doi:10.3389/fnhum.2022.815232)
Supplement: Supplementary file 1 [file Data_Sheet_1.docx]

Supplementary Material

**Table S1.** Patient profiles.

| Clinical information |  |
| --- | --- |
| Handedness | Right-handed |
| Age of onset (years) | 5 |
| Seizure Frequency | One to two times a year during the early time of disease, then it became more and more frequent. Although appropriate and adequate anti-epileptic drugs were used, the frequency of seizures was three to ten times per day prior surgery. |
| Duration of seizure | About ten to thirty seconds. |
| Putative epileptogenic zone | Left caudal dorsomedial prefrontal cortex |
| Epileptic symptom | Aura (including rustling of leaves, or voice of a young woman who was dressing in red skirt saying ‘what is the matter with you’) → chapeau de gendarme → automatic movement (hands) → hyperventilation |
| Antiepileptic drugstreatment | The patient had been taker Sodium Valproate,oxcarbazepine, Levetiracetam before surgery. |
| Intracranial surgerybefore SEEG implantation | No |
| Family history relevant toepilepsy | No |
| Intelligence evaluation | Wechsler Intelligence Scale, Full IntelligenceQuotient: 108Wechsler Memory Scale, Quotient：118 |
| Psychiatric disorder | No |

**Table S2.** Details of Electrical Stimulation Procedure and Subjective Reports.

| Target site | Brain Structure | BA Maps | Current (mA) | Patient Response |
| --- | --- | --- | --- | --- |
| Z9-Z10 | SFG | BA8 | 2.8 | “I could hear a young woman who was dressing in red skirt saying: what is the matter with you?”. The patient described that she had met this young woman when she was young and the woman said the same sentence to her. |
| F3-F4 | SFG | BA8 | 1.8 | “I could hear a young woman who was dressing in red skirt saying: what is the matter with you?”. The patient described that she had met this young woman when she was young and the woman said the same sentence to her. |
| T5-T6 | mSTG | BA22 | 2.4 | “I could hear abuzz voice in my head.” |
| T6-T7 | mSTG | BA22 | 2.0 | “I could hear abuzz voice in my right head.” |
| T7-T8 | mSTG | BA22 | 1.6 | “I could hear abuzz voice in my right head and ear.” |
| T8-T9 | mSTG | BA22 | 2.4 | “I could hear voice in my head.” |
| T9-T10 | mSTG | BA22 | 2.4 | “I could hear abuzz voice in my head.” |

BA: Brodmann area; mSTG: middle part of superior temporal gyrus; SFG: superior frontal gyrus.


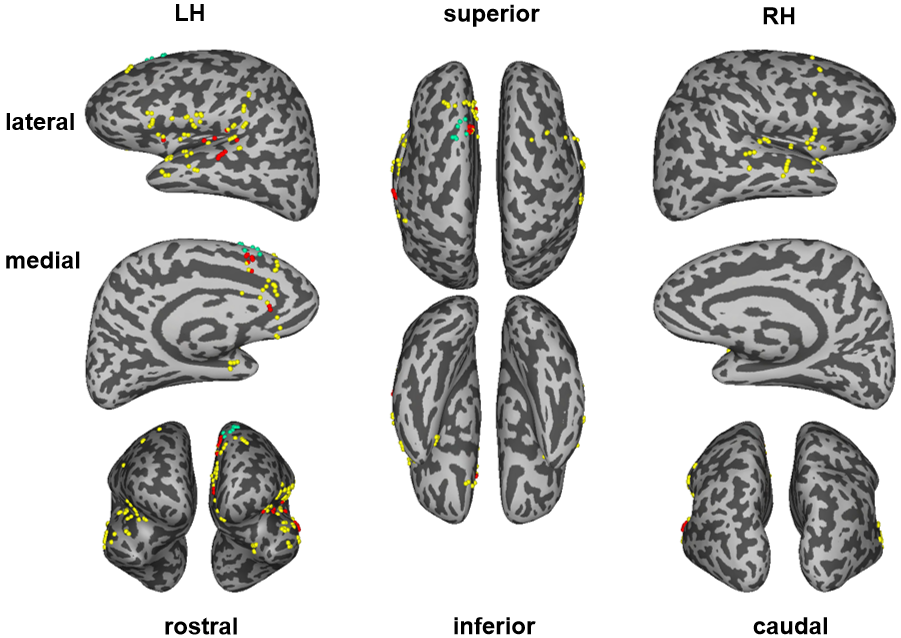


**Figure S1.** Anatomical locations of all the electrode sites in 6 views of left hemisphere (LH) and right hemisphere (RH). Note that the blue electrodes were in the epileptic zone, the red electrodes indicated electrodes that were close to the stimulation site (<15 mm) or bad recordings. Both the blue and red electrodes were excluded from analysis.

*
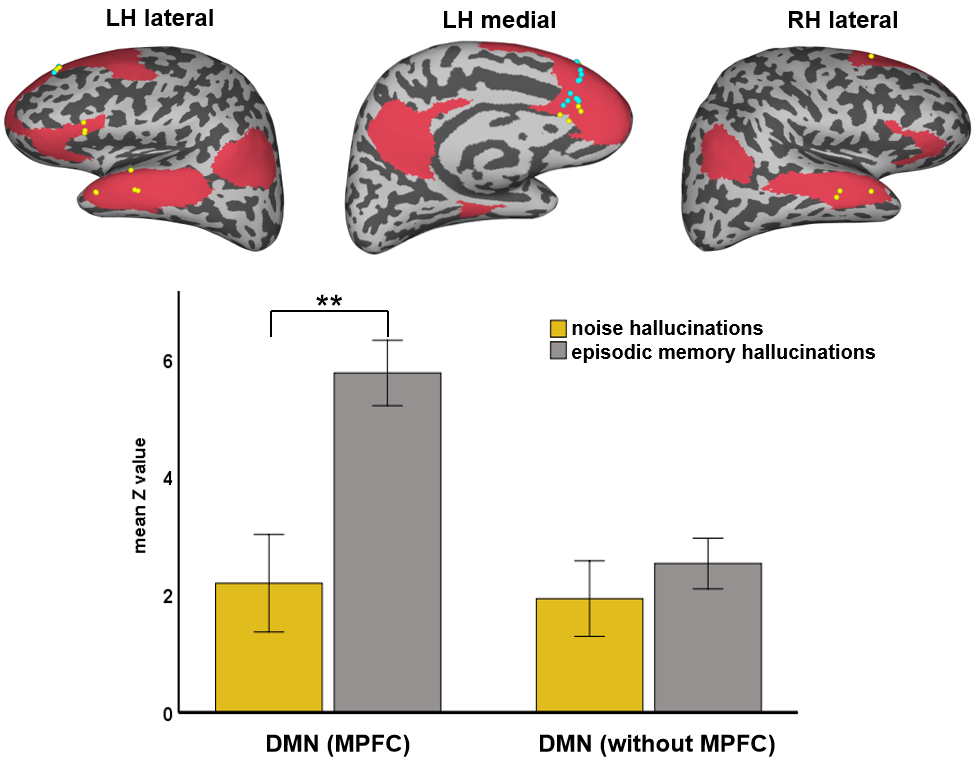
*

**Figure S2**. *Top panel*: Anatomical locations of electrodes in DMN (MPFC) and DMN (without MPFC) were showed. The red region represented the DMN, the blue electrodes indicated the electrodes in the MPFC and the yellow electrodes indicated the electrodes outside the MPFC. *Bottom panel*: Mean HGP Z values of two hallucination conditions in DMN (MPFC) and DMN (without MPFC). Error bars indicated ±1 SEM, **p<0.01.
